# Metastatic pathway and the microvascular and physicochemical microenvironments of human melanoma xenografts

**DOI:** 10.1186/s12967-017-1307-4

**Published:** 2017-10-10

**Authors:** Ruixia Huang, Lise Mari K. Andersen, Einar K. Rofstad

**Affiliations:** 1 Group of Radiation Biology and Tumor Physiology, Department of Radiation Biology, Institute for Cancer Research, Oslo University Hospital, Oslo, Norway; 2 Department of Radiation Biology, Institute for Cancer Research, Norwegian Radium Hospital, Box 4953, Nydalen, 0424 Oslo, Norway

**Keywords:** Vascular tumor microenvironment, Physicochemical tumor microenvironment, Melanoma, Metastasis, Hemangiogenesis, Lymphangiogenesis, Microvasculature, Interstitial fluid pressure, Hypoxia

## Abstract

**Background:**

Malignant melanoma of the skin can metastasize through blood vessels and lymphatics. The primary tumor develops a vascular microenvironment characterized by abnormal blood vessels and lymphatics and a physicochemical microenvironment characterized by low oxygen tension, regions with hypoxic tissue, and high interstitial fluid pressure (IFP). This study aimed at identifying relationships between the metastatic route of melanomas and characteristic features of the microvascular and physicochemical microenvironments of the primary tumor.

**Methods:**

Two patient-derived xenograft (PDX) models (E-13, N-15) and four cell line-derived xenografts (CDX) models (C-10, D-12, R-18, T-22) of human melanoma were included in the study. Tumors were transplanted to an orthotopic site in BALB/c-*nu*/*nu* mice, and when the tumors had grown to a volume of 500–600 mm^3^, the IFP of the primary tumor was measured and the hypoxia marker pimonidazole was administered before the host mouse was euthanized. The primary tumor, lungs, and six pairs of lymph nodes were evaluated by examining hematoxylin/eosin-stained and immunostained histological preparations. The expression of angiogenesis-related genes was assessed by quantitative PCR.

**Results:**

C-10, D-12, and E-13 tumors disseminated primarily by the hematogenous route and developed pulmonary metastases. These tumors showed high angiogenic activity and high expression of the F3 gene as well as ANGPT2 and TIE1, genes encoding proteins of the angiopoietin–tie system. N-15, R-18, and T-22 tumors disseminated mainly by the lymphogenous route and developed metastases in draining lymph nodes. These tumors had highly elevated IFP and showed high expression of NRP2, a gene encoding neuropilin-2.

**Conclusion:**

The primary metastatic route of orthotopic human melanoma xenografts and the development of lung and lymph node metastases are influenced significantly by the microvascular and physicochemical microenvironments of the primary tumor.

**Electronic supplementary material:**

The online version of this article (doi:10.1186/s12967-017-1307-4) contains supplementary material, which is available to authorized users.

## Background

Cutaneous melanoma is a highly aggressive disease of increasing incidence caused by malignant transformation of epidermal melanocytes [1, 2]. The disease progresses through distinct steps, and the transition from the radial growth phase to the vertical growth phase is associated with tumor-induced angiogenesis, increased metastatic propensity, and significant worsening of the prognosis [3, 4]. Primary melanomas in the vertical growth phase secret angiogenic factors that stimulate hem- and lymphangiogenesis, resulting in the development of a dense intratumoral network of blood vessels and an enriched peritumoral network of lymphatics [5–7]. High rates of hem- and lymphangiogenesis have been shown to be associated with metastatic growth and poor survival [8–13]. Recent reviews have concluded that the blood vessel density (BVD) in the tumor periphery and the peritumoral lymph vessel density (LVD) have significant prognostic power in patients with melanoma, whereas the BVD in central tumor regions and the intratumoral LVD may not have prognostic value [14–16].

Intravital microscopy studies have revealed that the blood vessel networks of melanomas in human patients show severe morphological abnormalities, including vessel disorganization, aberrant vessel bifurcations, heterogeneous vessel density, vessel tortuosity, increased vessel segment lengths, and highly permeable vessel walls [17]. These abnormalities cause irregular and heterogeneous blood flow, and may lead to the development of a physicochemical tumor microenvironment characterized by poor oxygenation and elevated interstitial fluid pressure (IFP) [18, 19]. Indeed, clinical investigations have revealed that low oxygen tension, hypoxic tumor regions, and high IFP are characteristic features of the physicochemical microenvironment of human melanomas [20–23].

Human melanoma xenografts are frequently being used as preclinical models of melanomas in patients, and it has been shown that the blood vessel and lymphatic networks of melanomas transplanted orthotopically to athymic nude mice are similar to those reported for human melanomas [24–26]. It has also been shown that orthotopic melanoma xenografts develop hypoxic regions and elevated IFP [27, 28], and furthermore, that metastatic dissemination and growth is associated with high angiogenic activity, extensive hypoxia, and high IFP in the primary tumor [29–31]. These findings have led to the suggestion that there is a link between the angiogenic signature, vascular morphology and function, and the physicochemical microenvironment in melanomas, and that interactions between these biological features may lead to a hostile tumor microenvironment promoting metastasis to regional lymph nodes and distant organs [32].

Cutaneous melanoma disseminates through blood vessels and lymphatics, and can show an organ specific metastatic pattern [33]. The time course to the development of distant metastases may differ between the metastatic pathways [34], and the progression of melanomas spreading by the hematogenous route is believed to be fast compared with that of melanomas spreading by the lymphogenous route [33–35]. Several genetic biomarkers associated with the metastatic route and organ specific metastasis have been identified [33]. However, studies examining the possibility that the metastatic pathway of melanomas is influenced significantly by the vascular and physicochemical tumor microenvironments are sparse.

In a previous study, we searched for associations between the overall metastatic propensity, the angiogenic potential, and the hypoxic tumor microenvironment of melanomas by using nine different melanoma xenograft lines as preclinical tumor models [36]. The study revealed that the metastatic propensity was determined by the tumor microvascular density rather than the fraction of hypoxic tissue, and vascular endothelial growth factor-A and interleukin-8 were identified as important drivers of tumor angiogenesis. In the study reported here, we addressed the possibility that the metastatic pathway and organ specific metastatic pattern of melanomas is influenced by the microvascular and physicochemical tumor microenvironments by searching for associations between metastatic spread and BVD, LVD, IFP, fraction of hypoxic tissue, and angiogenic signature. Orthotopic patient derived xenograft (PDX) models and cell line-derived xenograft (CDX) models of cutaneous melanoma were examined, and we provide significant evidence that tumor angiogenesis and the physicochemical tumor microenvironment may have strong impact on the metastatic pathway of melanomas.

## Methods

### Tumor models

Adult (8–12 weeks of age) female BALB/c *nu*/*nu* mice, bred and maintained at our research institute [32], were used as host animals for xenografted tumors. Four CDX models (C-10, D-12, R-18, T-22) and two PDX models (E-13, N-15) of metastatic melanoma were included in the study. The CDX models and the patients from whom permanent cell lines were established in culture have been described elsewhere [36]. The E-13 PDX model was obtained from a lymph node metastasis in the axilla of a 52-year-old female melanoma patient. The donor developed multiple pulmonary metastases and died from metastatic growth in the brain 18 months after presentation at the Norwegian Radium Hospital. The N-15 PDX model was derived from a lymph node metastasis in the abdomen of a 60-year-old male admitted to the Norwegian Radium Hospital for the treatment of metastatic melanoma. The donor patient responded poorly to chemotherapy and died from metastatic growth in the liver 27 months after the initiation of treatment. Both PDX models were established in BALB/c *nu*/*nu* mice by implanting small tumor pieces subcutaneously, and they have been maintained solely in vivo by serial subcutaneous transplantation of tumor cell aliquots. In the present study, we used orthotopic tumors initiated by intradermal inoculation of aliquots of ~ 3.5 × 10^5^ to ~ 1.0 × 10^6^ cells suspended in 10 μl of Hanks’ balanced salt solution. Tumors were included in experiments when having grown to a volume of 500–600 mm^3^. The volume (*V*) and the volume doubling time (*T*_d_) of the tumors were calculated as *V* = π/6 × *a* × *b* × *c* and *T*_d_ = ln2 × *t*/(ln*V*_t_ − ln*V*_0_), where *a*, *b*, and *c* are three orthogonal tumor diameters measured with calipers, and *V*_t_ and *V*_0_ are tumor volume at time *t* and time zero, respectively.

### Tumor interstitial fluid pressure

Tumor-bearing mice were anesthetized with fluanisone (20 mg/kg body weight), midazolam (10 mg/kg body weight), and fentanyl citrate (0.63 mg/kg body weight), and IFP was measured in the tumor center with a Millar SPC 320 catheter equipped with a 2F Mikro-Tip transducer (Millar Instruments, Houston, TX, USA) [28]. The catheter was connected to a computer, and data were acquired with the LabVIEW software. Two independent measurements were carried out in each tumor by inserting the IFP probe from two opposite directions, and these measurements provided highly similar IFP values.

### Immunohistochemical assessment of tumor hypoxic fraction, vessel density, and protein levels

Pimonidazole [1-[(2-hydroxy-3-piperidinyl)-propyl]-2-nitroimidazole], a marker of tumor hypoxia, was administered to the mice in a dose of 30 mg/kg body weight immediately after tumor IFP was measured. The mice were euthanized 3–4 h later, and the primary tumor was removed for immunohistochemical assessment of hypoxic fraction (HF_Pim_), vascular density, and the levels of angiopoietin-2, coagulation factor-III, tie1, and neuropilin-2. CD31 and LYVE-1 were used as markers of blood and lymph vessel endothelial cells, respectively. Histological sections were prepared by standard procedures, and immunostaining was carried out by using a peroxidase-based indirect staining method [29]. An anti-pimonidazole rabbit polyclonal antibody (provided by Professor Raleigh, University of North Carolina, Chapel Hill, NC, USA), an anti-mouse CD31 rabbit polyclonal antibody (Abcam, Cambridge, UK), an anti-mouse LYVE-1 rabbit polyclonal antibody (Abcam), an anti-human angiopoietin-2 rabbit polyclonal antibody (Abcam), an anti-human coagulation factor-III rabbit polyclonal antibody (Abcam), an anti-human tie1 rabbit polyclonal antibody (Abcam), or an anti-human neuropilin-2 rabbit polyclonal antibody (Abcam) was used as primary antibody. Diaminobenzidine was used as chromogen, and hematoxylin was used for counterstaining. Quantitative studies were carried out on preparations cut through the central regions of tumors and the surrounding tissue, and three sections of each staining were analyzed for each tumor. Staining densities were quantified by using the ImageJ software. Microvessels were defined and scored manually as described by Weidner [15]. Blood vessel density (BVD) in the invasive front (peripheral BVD) was assessed by counting vessels located within a 1-mm-thick band in the tumor periphery [32]. Peritumoral lymph vessel density (LVD) was determined by counting vessels in the surrounding skin located within a distance of 0.5 mm from the tumor surface [32]. HF_Pim_ was assessed by image analysis and was defined as the area fraction of the non-necrotic tissue showing positive pimonidazole staining [27].

### Tumor metastasis

The lungs and six pairs of lymph nodes (i.e., popliteal lymph nodes, inguinal lymph nodes, proper axillary lymph nodes, accessory axillary lymph nodes, medial iliac lymph nodes, and renal lymph nodes) were resected and examined for metastatic growth by light microscopy. Histological sections were cut at 100-μm intervals throughout the resected tissue and stained with hematoxylin and eosin. A group of five or more melanoma cells was scored as metastatic growth. Usually, the metastatic deposits had grown to a diameter of more than 100 μm when the lungs and lymph nodes were resected.

### Quantitative PCR (qPCR)

The RT^2^ Profiler PCR Array Human Angiogenesis (PAHS-024Z; SABiosciences, Frederick, MD, USA) was used for expression profiling of angiogenesis-related genes. The genes included in the array are presented in Additional file 1: Table S1. Total RNA was isolated from tumor tissue stabilized in RNA*later* RNA Stabilization Reagent (Qiagen, Hilden, Germany). RNA isolation, cDNA synthesis, and real-time qPCR were carried out as described earlier [26]. Fold difference in gene expression was calculated by using the ∆∆C_T_-method [37]. A C_T_-value of 35 (15 cycles above the positive PCR control) was set as detection limit, and consequently, genes with C_T_-values above 35 were not included in the analysis. The arrays included five house-keeping genes [β-actin (ACTB), β-2-microglobulin (B2M), glyceraldehyde-3-phosphate dehydrogenase (GAPDH), hypoxanthine phosphoribosyltransferase-1 (HPRT1), ribosomal protein lateral stalk subunit P0 (RPLP0)], and each C_T_-value of a tumor was normalized to the mean C_T_-value of these genes (∆C_T_ = C_T_^gene of interest^ − C_T_^mean of housekeeping genes^). Normalized gene expression levels were calculated from three tumors as $2^{{ - \,{\text{mean}}\;\Delta {\text{C}}_{\text{T}} }}$.

### Statistical analysis

Statistical analysis was carried out with the SigmaStat statistical software. Data are shown as mean ± standard error unless otherwise stated. The Kolmogorov–Smirnov method and the Levene’s method were used to test for normality and equal variance. Comparisons of data were carried out by using the Student *t* test (single comparisons) or by one-way ANOVA followed by the Bonferroni’s test (multiple comparisons) when the data complied with the conditions of normality and equal variance. Under other conditions, comparisons were carried out by non-parametric analysis using the Mann–Whitney rank-sum test (single comparisons) or by Kruskal–Wallis ANOVA on ranks followed by the Dunn’s test (multiple comparisons). Probability values of *p* < 0.05, determined from two-sided tests, were considered significant.

## Results

### The melanoma models showed different primary metastatic routes

**Fig. 1:**
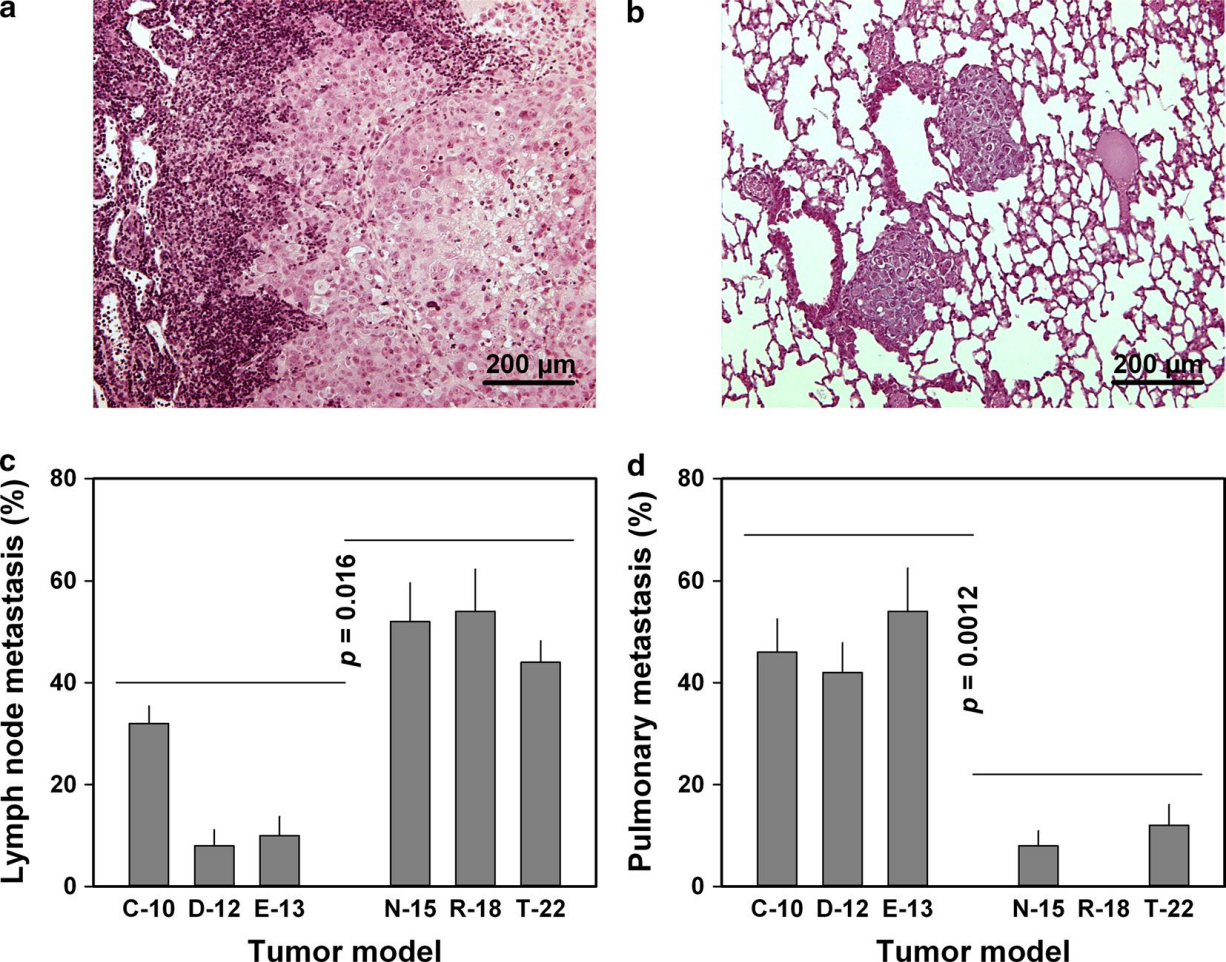
The metastatic pattern of six human melanoma xenograft models. Histological preparations showing metastatic growth in lymph node (**a**) and lung (**b**). C-10, D-12, and E-13 tumors showed lower incidence of lymph node metastasis (**c**) and higher incidence of pulmonary metastasis (**d**) than N-15, R-18, and T-22 tumors. The percentage of mice that showed metastatic growth was used as a parameter for incidence of metastasis. Columns and bars: mean values ± standard error of four experiments, each involving 10–15 mice per melanoma model. The experiments were carried out by using adult (8–12 weeks of age) female BALB/c *nu*/*nu* mice as host animals

Metastatic frequency was determined by studying a total of fifty tumor-bearing mice of each melanoma model. Four experiments were carried out, each involving 10–15 mice per model, and metastatic growth was observed frequently, both in lymph nodes (Fig. 1a) and lungs (Fig. 1b). The incidence metastasis (i.e., the percentage of mice that showed metastatic growth) and the metastatic pattern differed significantly among the six melanoma models, whereas the number of metastatic lesions in each metastasis-positive mouse did not. The N-15, R-18, and T-22 models showed higher incidence of lymph node metastasis than the C-10, D-12, and E-13 models (*p* = 0.016; Fig. 1c), whereas the incidence of pulmonary metastasis was higher in the C-10, D-12, and E-13 models than in the N-15, R-18, and T-22 models (*p* = 0.0012; Fig. 1d). Lymph node metastases develop primarily from tumor cells disseminated through lymphatics associated with the primary tumor, whereas the development of pulmonary metastases can be a result of tumor cell dissemination through primary tumor-associated blood vessels as well as tumor cell dissemination from lymph node metastases [6, 14, 16]. The incidence of lymph node metastasis was higher than the incidence of pulmonary metastases in the N-15, R-18, and T-22 models (*p* = 0.00075), and consequently, N-15, R-18, and T-22 tumors disseminated primarily through the lymphogenous route. The incidence of pulmonary metastasis was higher than the incidence of lymph node metastasis in the C-10, D-12, and E-13 models (*p* = 0.022), and consequently, indicative that the hematogenous route was the primary metastatic route in these tumors.

### The metastatic route differed with the physicochemical tumor microenvironment

**Fig. 2:**
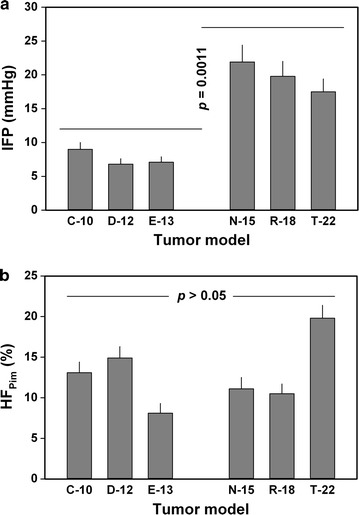
Tumor interstitial fluid pressure (IFP) and fraction of hypoxic tissue (HF_Pim_) in two groups of human melanoma xenografts differing in metastatic pattern. N-15, R-18, and T-22 tumors showed higher IFP than C-10, D-12, and E-13 tumors (**a**), but did not differ from C-10, D-12, and E-13 tumors in HF_Pim_ (**b**). Columns and bars: mean values ± standard error (n = 50 in **a** and n = 20 in **b**, where n represents the number of examined tumors). The experiments were carried out by using adult (8–12 weeks of age) female BALB/c *nu*/*nu* mice as host animals

IFP was measured in all primary tumors (i.e., 50 tumors per melanoma model), whereas 20 tumors of each model were selected randomly for immunostaining and assessment of HF_Pim_, BVD, and LVD. The N-15, R-18, and T-22 models showed higher IFP than the C-10, D-12, and E-13 models (*p* = 0.0011; Fig. 2a), suggesting an association between lymphogenous metastasis and highly elevated tumor IFP. HF_Pim_ did not differ between the C-10, D-12, and E-13 models on the one hand and the N-15, R-18, and T-22 models on the other, as illustrated qualitatively by using a C-10 and a T-22 tumor as representative examples (Additional file 2: Figure S1a) and shown quantitatively by presenting HF_Pim_ data for all six models (*p* > 0.05; Fig. 2b). This observation suggests that the primary metastatic route was not determined by the extent of tumor hypoxia.

### The metastatic route was linked to tumor angiogenic activity

**Fig. 3:**
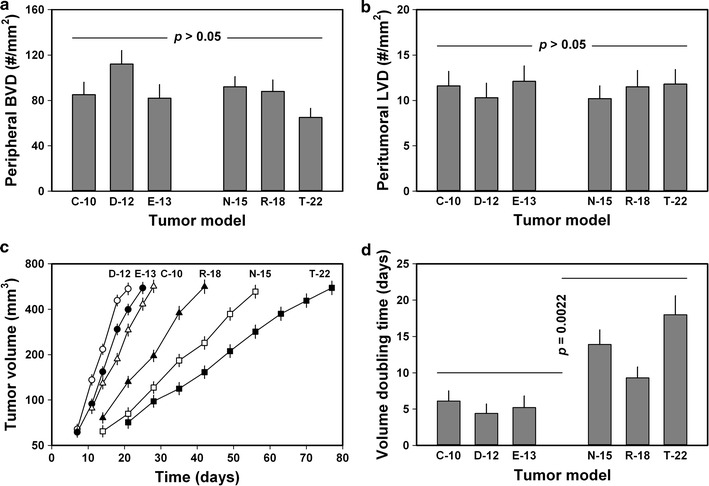
Peripheral blood vessel density (BVD), peritumoral lymph vessel density (LVD), tumor growth curves, and tumor volume doubling time (*T*_d_) of two groups of human melanoma xenografts differing in metastatic pattern. C-10, D-12, and E-13 tumors did not differ from N-15, R-18, and T-22 tumors in peripheral BVD (**a**) or peritumoral LVD (**b**), but grew faster (**c**) and, hence, had shorter *T*_d_ than N-15, R-18, and T-22 tumors (**d**). Columns and bars: mean values ± standard error (n = 20 in **a**, **b** and n = 50 in **d**, where n represents the number of studied tumors). Symbols and bars in **c**: Geometric mean ± standard error (n = 50). The experiments were carried out by using adult (8–12 weeks of age) female BALB/c *nu*/*nu* mice as host animals

The C-10, D-12, and E-13 models did not differ from the N-15, R-18, and T-22 models in peripheral tumor BVD or peritumoral LVD. This is illustrated qualitatively in Additional file 2: Figure S1b, c, which show representative examples of histological preparations immunostained for blood vessels and lymphatics, and quantitatively in Fig. 3a (*p* > 0.05) and Fig. 3b (*p* > 0.05). However, C-10, D-12, and E-13 tumors grew faster (*p* < 0.0001; Fig. 3c) and, hence, had shorter *T*_d_ (*p* = 0.0022; Fig. 3d) than N-15, R-18, and T-22 tumors. The rate of induction of new blood vessels (i.e., the angiogenic activity) is different in experimental tumors that show the same microvascular density but have different growth rates [38]. Because C-10, D-12, and E-13 tumors grew faster than N-15, R-18, and T-22 tumors and these two tumor groups had similar peripheral BVD and peritumoral LVD, the C-10, D-12, and E-13 models induced higher angiogenic activity than the N-15, R-18, and T-22 models. From these data and the data reported in Fig. 1, it follows that hematogenous metastatic dissemination and the development of pulmonary metastases were associated with high angiogenic activity in the primary tumor.

### The metastatic route was linked to tumor angiogenic signature

**Fig. 4:**
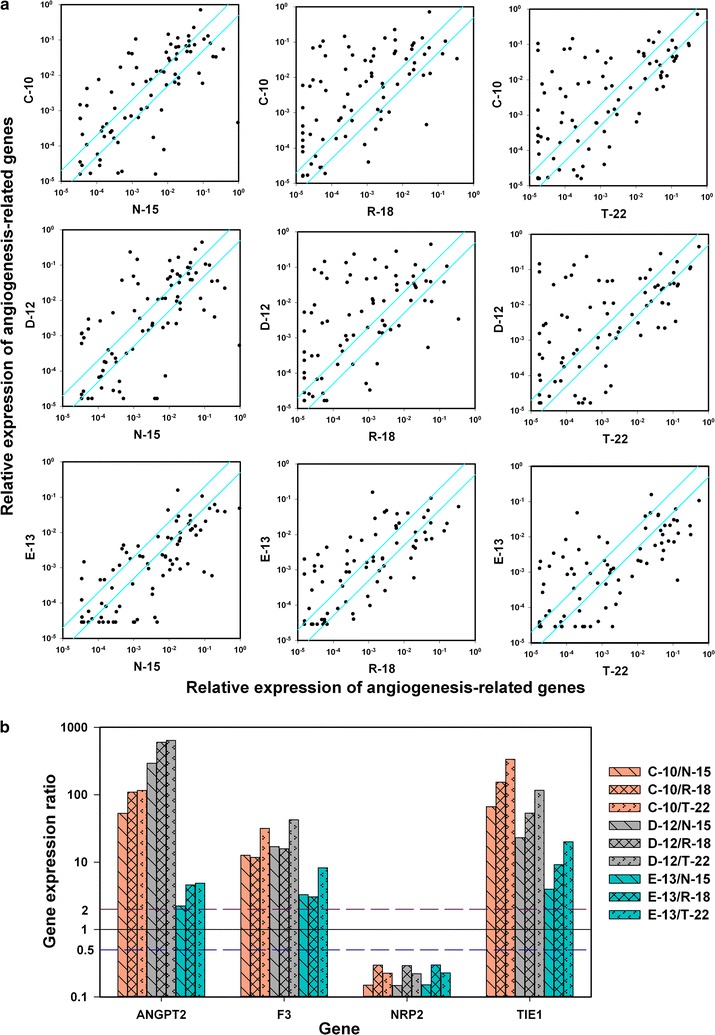
Expression of angiogenesis-related genes in two groups of human melanoma xenografts differing in metastatic pattern. A PCR array with 84 genes was used for expression profiling, and the genes on the array are presented in Additional file 1: Table S1. **a** Expression level in C-10, D-12, and E-13 tumors vs expression level in N-15, R-18, and T-22 tumors. Points: mean values of single genes determined from three different tumors. Solid lines: twofold difference in expression level. **b** Mean expression level of ANGPT2, F3, NRP2, and TIE1 in C-10, D-12, and E-13 tumors divided by that in N-15, R-18, and T-22 tumors. The mean expression of ANGPT2, F3, and TIE1 was more than twofold higher in C-10, D-12, and E-13 tumors than in N-15, R-18, and T-22 tumors [statistical analysis revealed that all 27 ratios were higher than 2 (*p* < 0.01)], whereas the mean expression of NRP2 was more than twofold higher in N-15, R-18, and T-22 tumors than in C-10, D-12, and E-13 tumors [statistical analysis revealed that all 9 ratios were lower than 0.5 (*p* < 0.005)]. The tumors were grown in adult (8–12 weeks of age) female BALB/c *nu*/*nu* mice

In general, more angiogenesis-related genes showed high expression in the C-10, D12, and E-13 models than in the N-15, R-18, and T-22 models (Fig. 4a). Three genes showed more than twofold higher expression in C-10, D-12, and E-13 tumors than in N-15, R-18, and T-22 tumors: ANGPT2 (angiopoietin-2), F3 (coagulation factor-III), and TIE1 (tyrosine kinase with immunoglobulin-like and epidermal growth factor-like domains-1), whereas NRP2 (neuropilin-2) was the only gene that showed more than twofold higher expression in N-15, R-18, and T-22 tumors than in C-10, D-12, and E-13 tumors (Fig. 4b).

**Fig. 5:**
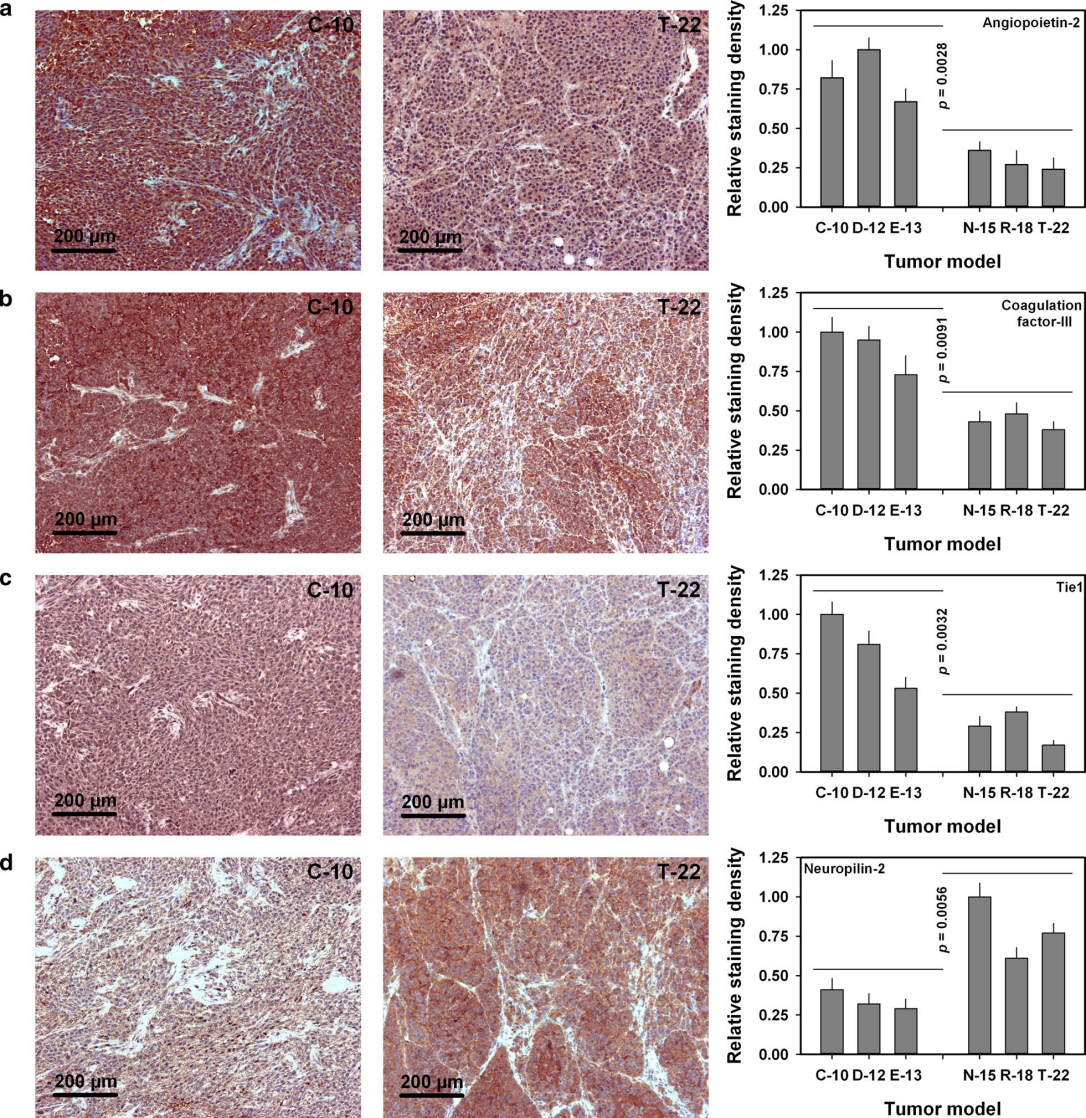
Immunohistochemical preparations. Images and staining densities of histological preparations immunostained for angiopoietin-2 (**a**), coagulation factor-III (**b**), tie1 (**c**), and neuropilin-2 (**d**). The images show representative examples, refer to a primary tumor of the C-10 melanoma xenograft model and a primary tumor of the T-22 melanoma xenograft model, and illustrate that C-10 tumors showed higher expression of angiopoietin-2, coagulation factor-III, and tie1 and lower expression of neuropilin-2 than T-22 tumors. Staining densities were for each protein normalized to the mean value of the tumor model that showed the highest staining density. C-10, D-12, and E-13 tumors showed higher staining density for angiopoietin-2, coagulation factor-III, and tie1 and lower staining density for neuropilin-2 than N-15, R-18, and T-22 tumors. Columns and bars: mean values ± standard error (n = 5, where n represents the number of studied tumors). The tumors were grown in adult (8–12 weeks of age) female BALB/c *nu*/*nu* mice

The protein levels of these genes were investigated by subjecting primary tumors and their metastases to immunohistochemistry. The immunohistochemistry data were fully in agreement with the qPCR data. The C-10, D-12, and E-13 models showed higher protein levels of the ANGPT2, F3, and TIE1 genes than the N-15, R-18, and T-22 models, whereas the NRP2 protein level was higher in the N-15, R-18, and T-22 models than in the C-10, D-12, and E-13 models, as illustrated in Fig. 5 by using a C-10 and a T-22 tumor as representative examples. Five tumors of each model were subjected to quantitative analysis, which revealed that the staining density of C-10, D-12, and E-13 tumors was higher than that of N-15, R-18, and T-22 tumors for angiopoietin-2 (*p* = 0.0028), coagulation factor III (*p* = 0.0091), and tie1 (*p* = 0.0032) and lower than that of N-15, R-18, and T-22 tumors for neuropilin-2 (*p* = 0.0056), as illustrated in Fig. 5. Thus, hematogenous metastatic spread and the development of pulmonary metastases were associated with high expression of ANGPT2, F3, and TIE1, whereas lymphogenous metastatic spread and the development of lymph node metastases were associated with high expression of NRP2.

## Discussion

Hematogenous and lymphogenous metastatic spread are characteristic features of angiogenic human melanomas [33, 34], and the possibility that the metastatic route is associated with the microvascular and physicochemical microenvironments of the primary tumor was investigated in this study by using orthotopic melanoma xenografts as preclinical models of human disease. C-10, D-12, and E-13 tumors disseminated primarily by the hematogenous route and developed pulmonary metastases, whereas N-15, R-18, and T-22 tumors mainly showed lymphogenous metastatic spread. These two tumor groups did not differ significantly in HF_Pim_, peripheral BVD, or peritumoral LVD, suggesting that tumor hypoxia and microvascular density are not important determinants of the primary metastatic route of melanomas.

Because the peripheral BVD of C-10, D-12, and E-13 tumors was similar to that of N-15, R-18, and T-22 tumors and C-10, D-12, and E-13 tumors grew faster and showed higher expression of angiogenesis-related genes than N-15, R-18, and T-22 tumors, the angiogenic activity was higher in C-10, D-12, and E-13 tumors than in N-15, R-18, and T-22 tumors. Consequently, hematogenous metastatic spread of melanomas may be associated with high angiogenic activity in the primary tumor. This suggestion is in accordance with clinical studies having shown higher angiogenic activity in the primary tumor in melanoma patients with distant organ metastases than in those with lymph node metastases only [39]. High angiogenic activity may promote hematogenous metastasis of tumors by several mechanisms. First, fast formation of new blood vessels leads to the development of an immature microvasculature with fragmented basement membrane, leaky vessels, and high density of vessel sprouts, morphological characteristics that may facilitate tumor cell intravasation [40]. Furthermore, elevated capacity to induce neovascularization may increase the probability of tumor cells trapped in secondary organ capillary beds to extravasate and give rise to macroscopic growth [41].

IFP was substantially higher in N-15, R-18, and T-22 tumors than in C-10, D-12, and R-18 tumors, suggesting that lymphogenous metastatic spread of melanomas is associated with highly elevated IFP in the central regions of the primary tumor. High IFP has been shown to promote lymph node metastasis also in human cervix carcinoma and pancreatic carcinoma xenografts [42, 43]. Mechanisms linking high IFP in tumors to lymph node metastasis have not been identified conclusively, but several possible mechanisms have been suggested. First, high tumor IFP may force interstitial fluid to flow from the tumor tissue into adjacent normal tissues [42], and this fluid flow may direct tumor cells toward peritumoral lymphatics by autologous chemotaxis [44]. Moreover, the interstitial fluid may transport proteolytic enzymes and chemokines that facilitate tumor cell migration by remodeling the extracellular matrix [44], and may carry lymphangiogenic factors that promote metastasis by dilating peritumoral lymphatics and inducing lymphangiogenesis [45].

Tumors develop elevated IFP because they show high resistance to blood flow, low resistance to transcapillary fluid flow, and impaired lymphatic drainage, and the intertumor heterogeneity in IFP is primarily a consequence of differences in blood flow resistance [46]. Low-diameter vessels are the main cause of high resistance to blood flow, but abnormal vessel bifurcations, vessel tortuosity, and long vessel segment lengths may also contribute significantly [47]. The resistance to blood flow was most likely higher in N-13, R-18, and T-22 tumors than in C-10, D-12, and E-13 tumors, resulting in higher IFP in the former group of tumors. Taken together, our observations suggest that melanomas disseminating primarily by the lymphogenous route develop a microvascular network that exerts high resistance to blood flow and causes highly elevated IFP, whereas melanomas disseminating primarily by the hematogenous route have highly elevated angiogenic activity that results in a microvascular network with dilated vessels that exerts lower resistance to blood flow and facilitates tumor cell intravasation.

The expression of ANGPT2, F3, and TIE1 was more than twofold higher in C-10, D-12, and E-13 tumors than in N-15, R-18, and T-22 tumors, and the expression of NRP2 was more than twofold higher in N-15, R-18, and T-22 tumors than in C-10, D-12, and E-13 tumors. These observations suggest that high expression of F3 and genes of the angiopoietin–tie system is associated with high angiogenic activity, hematogenous metastatic spread, and the development of pulmonary metastases in melanoma xenografts, whereas high expression of NRP2 is associated with lymphogenous metastatic spread and the development of lymph node metastases. Further studies are needed to ascertain whether there is a causal relationship between elevated expression of these genes and the metastatic route of melanomas.

The angiopoietin–tie system plays important roles in vascular development, morphogenesis, and homeostasis, and is implicated in several diseases where the vasculature is important, including cancer [48, 49]. Blocking of ANGPT2 protein or genetic deletion of TIE1 has been shown to decrease tumor angiogenesis and growth by reducing endothelial cell sprouting and by inducing endothelial cell apoptosis and vessel regression [50, 51]. It has also been revealed that blocking of ANGPT2 protein may induce vascular normalization in tumors [52], inhibit transendothelial migration of tumor cells [53], and decrease pulmonary metastasis and growth of experimental tumors [54–56]. The association between hematogenous metastatic spread of melanomas and high expression of ANGPT2 and TIE1 reported here is consistent with these observations.

F3 encodes coagulation factor III, also known as tissue factor (TF). Full length TF promotes tumor angiogenesis by up-regulating the expression of several proangiogenic factors, including interleukin-8, chemokine (C-X-C motif) ligand-1, and vascular endothelial growth factor-A [57, 58]. Spliced TF may stimulate tumor angiogenesis through protein tyrosine kinase-2 signaling by ligating to endothelial cell integrins such as αvβ3 and α6β1 [57, 59]. In addition, TF may play an important role in tumor cell intravasation [60]. The significance of TF in lung metastasis of melanoma has been studied previously [61, 62]. TF was found to promote lung colonization of melanoma cells inoculated intravenously into immunodeficient mice, but did not facilitate spontaneous lung metastasis of subcutaneous melanomas. It is thus possible that the association between pulmonary metastasis and high expression of F3 reported here was due to effects of TF in the later phases of the metastatic process after the melanoma cells had entered the blood circulation as well as effects of TF within the primary tumor.

NRP2 encodes the neuropilin-2 protein, which is a transmembrane multifunctional nonkinase receptor for class III semaphorins, members of the vascular endothelial growth factor family, and other growth factors [63]. Tumor-induced activation of neuropilin-2 on lymphatic endothelial cells can increase peritumoral lymphangiogenesis and promote lymph node metastasis [64]. Neuropilin-2 on tumor cells interacts with integrins on blood and lymph vessel endothelial cells to mediate vascular adhesion and promote extravasation [65]. High expression of NRP2 has been shown to promote hematogenous metastatic spread in pancreatic adenocarcinoma and clear cell renal cell carcinoma xenografts [65] and the development of lymph node metastases in patients with breast carcinoma [66], papillary thyroid carcinoma [67], and squamous cell carcinoma of the oesophagus [68]. Immunohistochemical investigations have revealed that melanomas show high expression of neuropilin-2 and that the expression is higher in lymph node metastases than in the primary tumor [69]. These observations suggest that the association between high expression of NRP2 and lymph node metastasis reported here was not due to effects of neuropilin-2 within the primary tumor, but most likely was due to interaction between melanoma cells and lymphatic endothelial cells after the melanoma cells had entered the peritumoral lymphatic network.

## Conclusions

Orthotopic melanoma xenografts can metastasize by the hematogenous and the lymphogenous pathway. Melanomas that disseminate primarily through the hematogenous route show high angiogenic activity in the primary tumor, high incidence of pulmonary metastases, and high expression of the F3 gene as well as genes encoding central proteins of the angiopoietin–tie system. Melanomas that spread primarily through the lymphogenous route are characterized by highly elevated IFP in the primary tumor and metastatic growth in draining lymph nodes. High IFP is mainly a consequence of high resistance to blood flow, and metastatic growth in lymph nodes is most likely facilitated by high expression of NRP2.

## Additional files

**Additional file 1.** Angiogenesis-related genes included in the PCR array.

**Additional file 2.** Histological preparations of a representative C-10 tumor and a representative T-22 tumor immunostained for pimonidazole to visualize tumor hypoxia, immunostained for CD31 to visualize tumor blood vessels, and immunostained for LYVE-1 to visualize peritumoral lymphatics in skin.

## Abbreviations

ACTB: β-actin
ANGPT2: angiopoietin-2
B2M: β-2-microglobulin
BVD: blood vessel density
CDX models: cell line-derived xenograft models
F3: coagulation factor-III
GAPDH: glyceraldehyde-3-phosphate dehydrogenase
HF_Pim_: hypoxic fraction assessed with pimonidazole as hypoxia marker
HPRT1: hypoxanthine phosphoribosyltransferase-1
IFP: interstitial fluid pressure
LVD: lymph vessel density
NRP2: neuropilin-2
PDX models: patient-derived xeno-graft models
qPCR: quantitative polymerase chain reaction
RPLP0: ribosomal protein lateral stalk subunit P0
*T*_d_: tumor volume doubling time
TF: tissue factor
TIE1: tyrosine kinase with immunoglobulin-like and epidermal growth factor-like domains-1
