# Supplementary material for: Metastatic pathway and the microvascular and physicochemical microenvironments of human melanoma xenografts
Source: J Transl Med. 2017 Oct 10;15:203. doi: 10.1186/s12967-017-1307-4 (PMC5634823; doi:10.1186/s12967-017-1307-4)
Supplement: Supplementary file 1 — Additional file 1. Angiogenesis-related genes included in the PCR array. [file 12967_2017_1307_MOESM1_ESM.pdf]

Additional file 1: Table S1 to:

Metastatic pathway and the microvascular and physicochemical microenvironments of human melanoma xenografts

Ruixia Huang[1], Lise Mari K. Andersen[1] and Einar K. Rofstad[1*]

[1]Group of Radiation Biology and Tumor Physiology, Department of Radiation Biology, Institute for Cancer Research, Oslo University Hospital, Oslo, Norway

E-mail addresses:

Ruixia Huang (ruixia.huang@rr-research.no)

Lise Mari K. Andersen (lise.mari.klepp.andersen@rr-research.no)

Einar K. Rofstad (einar.k.rofstad@rr-research.no)

* Correspondence: Einar K. Rofstad, Department of Radiation Biology, Institute for Cancer Research, Norwegian Radium Hospital, Box 4953 Nydalen, 0424 Oslo, Norway.
Phone: 47-2278-1206; Fax: 47-2278-1207; E-mail: einar.k.rofstad@rr-research.no

Additional file 1: Table S1. Angiogenesis-related genes included in the PCR array*

Gene Refseq**	Symbol	Name
NM_005163	AKT1	V-akt murine thymoma viral oncogene homolog 1
NM_001145	ANG	Angiogenin. ribonuclease. RNase A family. 5
NM_001146	ANGPT1	Angiopoietin 1
NM_001147	ANGPT2	Angiopoietin 2
NM_001039667	ANGPTL4	Angiopoietin-like 4
NM_001150	ANPEP	Alanyl (membrane) aminopeptidase
NM_001702	BAI1	Brain-specific angiogenesis inhibitor 1
NM_002986	CCL11	Chemokine (C-C motif) ligand 11
NM_002982	CCL2	Chemokine (C-C motif) ligand 2
NM_001795	CDH5	Cadherin 5. type 2 (vascular endothelium)
NM_030582	COL18A1	Collagen. type XVIII. alpha 1
NM_000091	COL4A3	Collagen. type IV. alpha 3 (Goodpasture antigen)
NM_001901	CTGF	Connective tissue growth factor
NM_001511	CXCL1	Chemokine (C-X-C motif) ligand 1 (melanoma growth stimulating activity. alpha)
NM_001565	CXCL10	Chemokine (C-X-C motif) ligand 10
NM_002994	CXCL5	Chemokine (C-X-C motif) ligand 5
NM_002993	CXCL6	Chemokine (C-X-C motif) ligand 6 (granulocyte chemotactic protein 2)
NM_002416	CXCL9	Chemokine (C-X-C motif) ligand 9
NM_001955	EDN1	Endothelin 1
NM_182685	EFNA1	Ephrin-A1
NM_004093	EFNB2	Ephrin-B2
NM_001963	EGF	Epidermal growth factor
NM_000118	ENG	Endoglin
NM_004444	EPHB4	EPH receptor B4
NM_004448	ERBB2	V-erb-b2 receptor tyrosine kinase 2
NM_001993	F3	Coagulation factor III (thromboplastin. tissue factor)
NM_000800	FGF1	Fibroblast growth factor 1 (acidic)
NM_002006	FGF2	Fibroblast growth factor 2 (basic)
NM_000142	FGFR3	Fibroblast growth factor receptor 3
NM_004469	FIGF	C-fos induced growth factor (vascular endothelial growth factor D)
NM_002019	FLT1	Fms-related tyrosine kinase 1 (VEGFR1)
NM_002026	FN1	Fibronectin 1
NM_000601	HGF	Hepatocyte growth factor (hepapoietin A; scatter factor)
NM_001530	HIF1A	Hypoxia inducible factor 1. alpha subunit (basic helix-loop-helix transcription factor)
NM_006665	HPSE	Heparanase
NM_002165	ID1	Inhibitor of DNA binding 1. dominant negative helix-loop-helix protein
NM_024013	IFNA1	Interferon. alpha 1
NM_000619	IFNG	Interferon. gamma
NM_000618	IGF1	Insulin-like growth factor 1 (somatomedin C)
NM_000576	IL1B	Interleukin 1. beta
NM_000600	IL6	Interleukin 6 (interferon. beta 2)
NM_000584	IL8	Interleukin 8
NM_002210	ITGAV	Integrin. alpha V (vitronectin receptor. alpha polypeptide. antigen CD51)
NM_000212	ITGB3	Integrin. beta 3 (platelet glycoprotein IIIa. antigen CD61)
NM_000214	JAG1	Jagged 1
NM_002253	KDR	Kinase insert domain receptor (a type III receptor tyrosine kinase)
NM_007015	LECT1	Leukocyte cell derived chemotaxin 1

NM_000230	LEP	Leptin
NM_002391	MDK	Midkine (neurite growth-promoting factor 2)
NM_004995	MMP14	Matrix metallopeptidase 14 (membrane-inserted)
NM_004530	MMP2	Matrix metallopeptidase 2 (gelatinase A. 72kDa gelatinase. 72kDa type IV collagenase)
NM_004994	MMP9	Matrix metallopeptidase 9 (gelatinase B. 92kDa gelatinase. 92kDa type IV collagenase)
NM_000603	NOS3	Nitric oxide synthase 3 (endothelial cell)
NM_004557	NOTCH4	Notch 4
NM_003873	NRP1	Neuropilin 1
NM_003872	NRP2	Neuropilin 2
NM_002607	PDGFA	Platelet-derived growth factor alpha polypeptide
NM_000442	PECAM1	Platelet/endothelial cell adhesion molecule
NM_002619	PF4	Platelet factor 4
NM_002632	PGF	Placental growth factor
NM_002658	PLAU	Plasminogen activator. urokinase
NM_000301	PLG	Plasminogen
NM_021935	PROK2	Prokineticin 2
NM_000962	PTGS1	Prostaglandin-endoperoxide synthase 1 (prostaglandin G/H synthase and cyclooxygenase)
NM_001400	S1PR1	Sphingosine-1-phosphate receptor 1
NM_000602	SERPINE1	Serpin peptidase inhibitor. clade E (nexin. plasminogen activator inhibitor type 1). member 1
NM_002615	SERPINF1	Serpin peptidase inhibitor. clade F (alpha-2 antiplasmin. pigment epithelium derived factor). member 1
NM_021972	SPHK1	Sphingosine kinase 1
NM_000459	TEK	TEK tyrosine kinase. endothelial
NM_003236	TGFA	Transforming growth factor. alpha
NM_000660	TGFB1	Transforming growth factor. beta 1
NM_003238	TGFB2	Transforming growth factor. beta 2
NM_004612	TGFBR1	Transforming growth factor. beta receptor 1
NM_003246	THBS1	Thrombospondin 1
NM_003247	THBS2	Thrombospondin 2
NM_005424	TIE1	Tyrosine kinase with immunoglobulin-like and EGF-like domains 1
NM_003254	TIMP1	TIMP metallopeptidase inhibitor 1
NM_003255	TIMP2	TIMP metallopeptidase inhibitor 2
NM_000362	TIMP3	TIMP metallopeptidase inhibitor 3
NM_000594	TNF	Tumor necrosis factor
NM_001953	TYMP	Thymidine phosphorylase
NM_003376	VEGFA	Vascular endothelial growth factor A
NM_003377	VEGFB	Vascular endothelial growth factor B
NM_005429	VEGFC	Vascular endothelial growth factor C

* Human angiogenesis RT² Profiler™ PCR array (Catalog number PAHS-024Z, SABiosciences/Qiagen). **NCBI reference sequence database.
