# Supplementary material for: Metastatic pathway and the microvascular and physicochemical microenvironments of human melanoma xenografts
Source: J Transl Med. 2017 Oct 10;15:203. doi: 10.1186/s12967-017-1307-4 (PMC5634823; doi:10.1186/s12967-017-1307-4)
Supplement: Supplementary file 2 — Additional file 2. Histological preparations of a representative C-10 tumor and a representative T-22 tumor immunostained for pimonidazole to visualize tumor hypoxia, immunostained for CD31 to visualize tumor blood vessels, and immunostained for LYVE-1 to visualize peritumoral lymphatics in skin. [file 12967_2017_1307_MOESM2_ESM.pdf]

Additional file 2: Figure S1 to:

Metastatic pathway and the microvascular and physicochemical microenvironments of human melanoma xenografts

Ruixia Huang[1], Lise Mari K. Andersen[1] and Einar K. Rofstad[1*]

[1]Group of Radiation Biology and Tumor Physiology, Department of Radiation Biology, Institute for Cancer Research, Oslo University Hospital, Oslo, Norway

E-mail addresses:

Ruixia Huang (ruixia.huang@rr-research.no)

Lise Mari K. Andersen (lise.mari.klepp.andersen@rr-research.no)

Einar K. Rofstad (einar.k.rofstad@rr-research.no)

* Correspondence: Einar K. Rofstad, Department of Radiation Biology, Institute for Cancer Research, Norwegian Radium Hospital, Box 4953 Nydalen, 0424 Oslo, Norway.
Phone: 47-2278-1206; Fax: 47-2278-1207; E-mail: einar.k.rofstad@rr-research.no

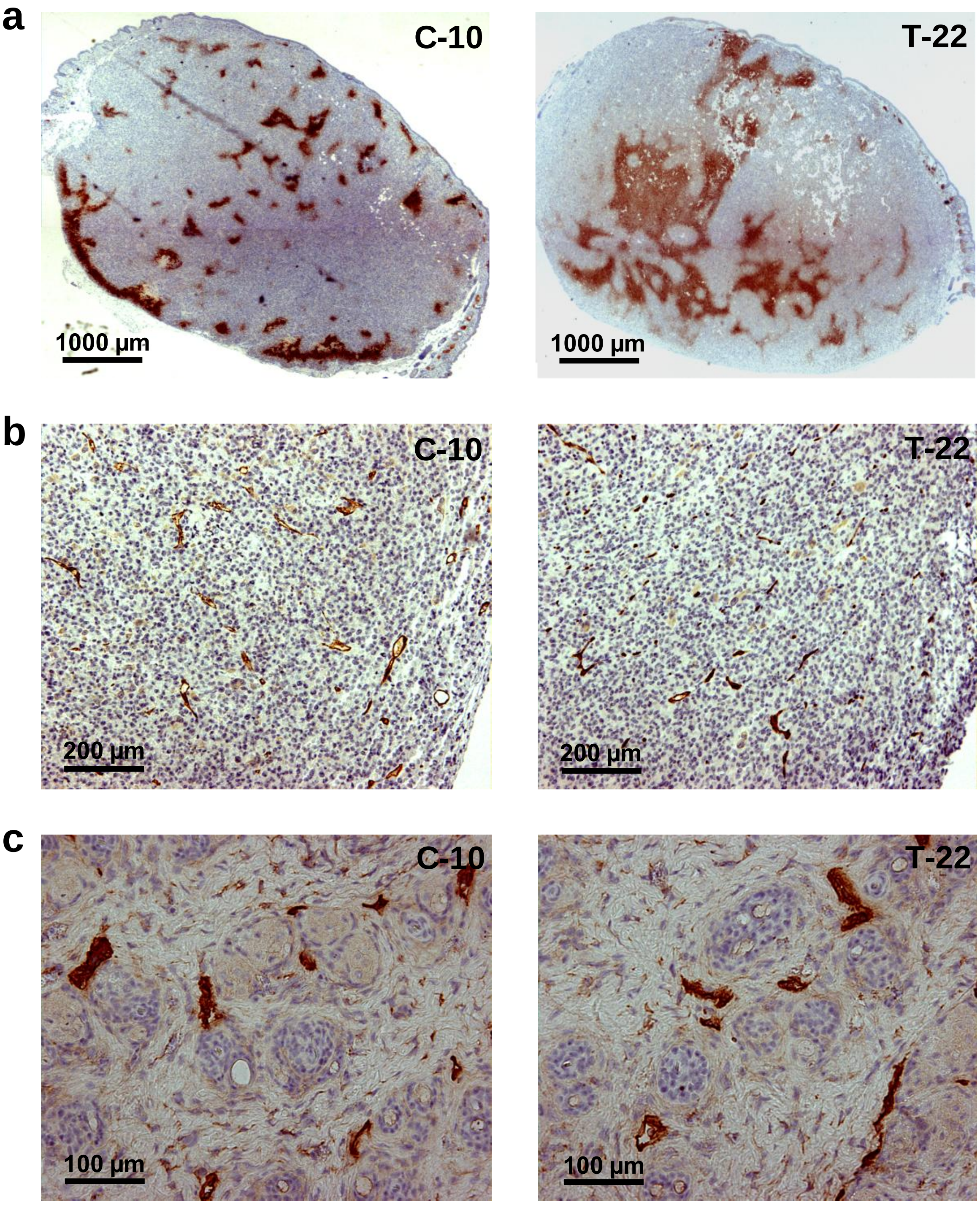

Additional file 2: Figure S1 Immunohistochemical preparations. Histological preparations of a reprecentative C-10 tumor and a representative T-22 tumor immunostained for pimonidazole to visualize hypoxia (**a**), immunostained for CD31 to visualize tumor blood vessels (**b**), and immunostained for LYVE-1 to visualize peritumoral lymphatics in skin (**c**). The images show the general quality of the preparations used for assessment of fraction of hypoxic tumor tissue, peripheral tumor blood vessel density, and peritumoral lymph vessel density in C-10, D-12, E-13, N-15, R-18, and T-22 melanoma xenografts grown in adult (8–12 weeks of age) female BALB/c *nu/nu* mice. The C-10, D-12, and E-13 models did not differ from the N-15, R-18, and T-22 models in fraction of hypoxic tumor tissue, peripheral tumor blood vessel density, or peri-tumoral lymph vessel density, as illustrated qualitatively by using a C-10 and a T-22 tumor as examples.
